# Association of a missense mutation in the luteinizing hormone/choriogonadotropin receptor gene (*LHCGR*) with superovulation traits in Chinese Holstein heifers

**DOI:** 10.1186/2049-1891-3-35

**Published:** 2012-11-09

**Authors:** Yong Yu, Yunwei Pang, Haichao Zhao, Xiaoling Xu, Zhonghong Wu, Lei An, Jianhui Tian

**Affiliations:** 1 Key Laboratory of Animal Genetics and Breeding of the Ministry of Agriculture, College of Animal Science and Technology, China Agricultural University, Beijing, 100193, PR China

**Keywords:** Chinese Holstein heifer, LHCGR, SNP, Superovulation

## Abstract

**Background:**

Upon binding luteinizing hormone in the ovary, the luteinizing hormone/choriogonadotropin receptor (LHCGR) is necessary for follicular maturation and ovulation, as well as luteal function. We detected mutations in the LHCGR gene and evaluated their association with superovulation.

**Methods:**

Using polymerase chain reaction-single strand conformation polymorphism (PCR-SSCP) and DNA sequencing, we examined polymorphisms in *LHCGR* and the genotypes associated with superovulation traits in 127 Chinese Holstein heifers.

**Results:**

A G/T polymorphism (ss52050737) in exon 11 was significantly associated with the total number of ova and the number of transferable embryos.

**Conclusions:**

*LHCGR* may be a new predictor for superovulation in Chinese Holstein heifers.

## Background

In cattle, the pituitary gonadotropin luteinizing hormone (LH) plays a role in follicular development [1], ovulation [1,2], corpora lutea formation [3], and preimplantation embryonic development [4,5]. The cellular actions of LH are mainly mediated by the luteinizing hormone/choriogonadotropin receptor (LHCGR), which has features typical of receptors that interact with G proteins, including a cellular domain, seven transmembrane domains, and an extracellular hormone-binding domain [6]. LHR cDNAs have been cloned in several species [7-10], and several mRNA variants have been reported [11-15]. In cattle, LHR splice variants have been reported with deletions of exon 10 and partial deletions of exon 11 or exon 3 [14,15]. The former two variants are found significantly more frequently in cultured granulosa cells compared with cells from dominant follicles [14].

The technologies for achieving multiple ovulation and embryo transfer (MOET) are well established, and more than 500,000 embryos are produced annually from superovulated cows worldwide [16]. However, variability in the superovulation response among individuals continues to be one of the most frustrating problems with respect to embryo transfer in cattle [17]. Although great progress has been made toward understanding folliculogenesis and regulation of the hypothalamic-pituitary-gonadal axis, and many approaches to superovulatory treatments have been explored, the high variability among individuals in ovarian responsiveness to the application of exogenous hormones remains a challenge. One reason for this variability is that superovulation traits are quantitative and are strongly affected by the environment. Adjustments to superovulation protocols related to luteinizing hormone/choriogonadotropin receptor (LHCGR) availability may increase the yield of bovine embryos [18]. The follicle stimulating hormone receptor (*FSHR*), inhibin alpha (*INHA*) and progesterone receptor (*PGR*) genes have been reported as predictors of superovulation in Chinese Holstein cows [19-21]. In addition, three single-nucleotide polymorphisms (SNPs) in exon 11 of the *LHCGR* gene are associated with bovine fertility traits [22], and four SNPs of G51656T, A51703G, A51726G and G51737A in intron 9 of the *LHCGR* gene had significant effects on the total number of ova (TNO) recovered from superovulated Holstein cows [23]. Therefore, *LHCGR* was examined further as a candidate gene for predicting response to superovulation in Chinese Holstein heifers. The objective of this study was to investigate the prognostic significance of *LHCGR* genotypes in superovulation responsiveness.

## Methods

### Experimental animals and sampling

A total of 127 Chinese Holstein heifers from 60.6 to 68.7 week of age old were obtained from four breeding farms of Beijing Dairy Cattle Center and used for superovulation treatment (see below). Blood samples were collected form each heifer aseptically from the caudal vein into a tube containing EDTA (ethylene diamine tetraacetic acid) as an anticoagulant. Genomic DNA was extracted using the RelaxGene Blood DNA System (Tiangen, Biotech, Co. Ltd.) and stored at −20°C.

### Superovulation and embryo harvest

Superovulation was induced throughout the year except for the months of June, July and August using a 4-day regimen of decreasing doses (i.e. dose of 75, 75, 50, 50, 30, 30, 10 and 10 mg for each injection) of follicle stimulating hormone (Folltropin-V; Vetrepharm Canada Inc. , Belleville, ON, Canada) given at 12 h intervals beginning on day 4 after insertion of an intravaginal progesterone-releasing device (Day 0; CIDR, Inter Ag). A dose of 0.4 mg Prostaglandin F2α (Shanghai Institute of Planned Parenthood Research; SIPPR, Shanghai, China) was given to each heifer on the morning of day 6, and the CIDR device was removed that afternoon. Estrus was detected visually on the morning of day 8. At approximately 7:00 p.m. on day 8, each heifer was randomly artificially inseminated (AI) with the first of two straws from one of eight bulls containing sexed frozen-thawed sperm. AI with the second straw was performed 12 h later. On day 16, each uterine horn was flushed non-surgically with 500 ml Dulbecco’s phosphate-buffered saline and the recovered fluid was examined for oocytes or embryos under a stereomicroscope. Embryos were isolated and classified as transferable embryos or degenerating embryos according to the criteria of the International Embryo Transfer Society (IETS). Oocytes were defined as unfertilized ova. Heifers with no oocytes or embryos were defined as non-responders.

### Primer design and PCR amplification

Primer set 1 (sense: 5^′^-CTGAGTGGCTGGGATTAT-3^′^; anti-sense: 5^′^-CGGGAGGGCTTATTTGAT-3^′^) was designed to amplify exon 11 of the *LHCGR* [accession: ID281900] gene. Primer set 2 (sense: 5^′^-GCTCTACCTGCTGCTCAT-3^′^; anti-sense: 5^′^-TAATTGCTGACACCCACA-3^′^) was designed for genotyping the detected SNP (ss52050737). PCR was performed in a 20 μL reaction containing 0.1 μm/L each primer, 4 μm/L dNTPs (deoxyribonucleoside triphosphates), 2 μL 10× PCR buffer (containing Mg^2+^), 0.5 U TaKaRa Taq polymerase (TaKaRa Biotechnology, Co. Ltd.), and 50 ng genomic DNA as template. PCR reactions consisted of denaturation at 95°C for 5 min, followed by 35 cycles of 95°C for 30 sec, annealing for 30 sec at 57°C for primer set 1 and primer set 2, and extension at 72°C for 70 sec for primer set 1 and 30 sec for primer set 2, followed by a final extension at 72°C for 5 min. PCR products were analyzed with 1% agarose gel electrophoresis in TAE buffer (0.89 mol/L Tris, 0.02 mol/L Na_2_EDTA, 0.89 mol/L boric acid) and stained with ethidium bromide.

### DNA sequencing and genotyping

Amplified products from individual samples using primer set 1 were mixed and sequenced commercially (Invitrogen Beijing Office, Beijing, China). The mutation detected was genotyped by PCR-SSCP using primer set 2. PCR products (3 μL) were mixed with 8 μL denaturing solution (95% formamide, 25 mmol/L EDTA, 0.025% xylene-cyanol, 0.025% bromophenol blue), heated for 10 min at 98°C, and chilled on ice. Denatured DNA was subjected to 13% polyacrylamide gel electrophoresis (PAGE) (39:1 acrylamide/bisacrylamide) in 1× Tris-borate EDTA (TBE) buffer at constant voltage (120 V) for 8–10 h. The gel was stained with 0.1% silver nitrate, and products with different electrophoresis patterns were sequenced in both directions.

### Statistical analysis

Association of the genotype of the mutation with superovulation traits was calculated using the General Linear Model of PASW Statistics 18 (SPSS Inc. , Chicago, Illinois, USA). The factors considered were genotype, yard, age, season, and bull. The statistical model was

(1) $$Y_{\mathrm{jkhi}}\;=u\;+\;G_{j}\;+A_{k}\;+\;S_{h}\;+\;D_{i}\;+\;B_{l}\;+\;e_{\mathrm{jkhil}}$$

where *Y*_jkhi_ was the phenotypic value of traits, *u* was the population mean, *G*_j_ was the fixed effect of genotypes, *A*_k_ was the effect of age and acted as a covariate, *S*_h_ was the fixed effect of season (spring, autumn and winter), *D*_i_ was the effect of the yard, *B*_l_ was the effect of the bull and acted as a random variable, and *e*_jkhil_ was the random residual error. For the TNO, the factor of the bull was removed from the model.

## Results

### Genotypic and allelic frequencies

**Figure 1:**
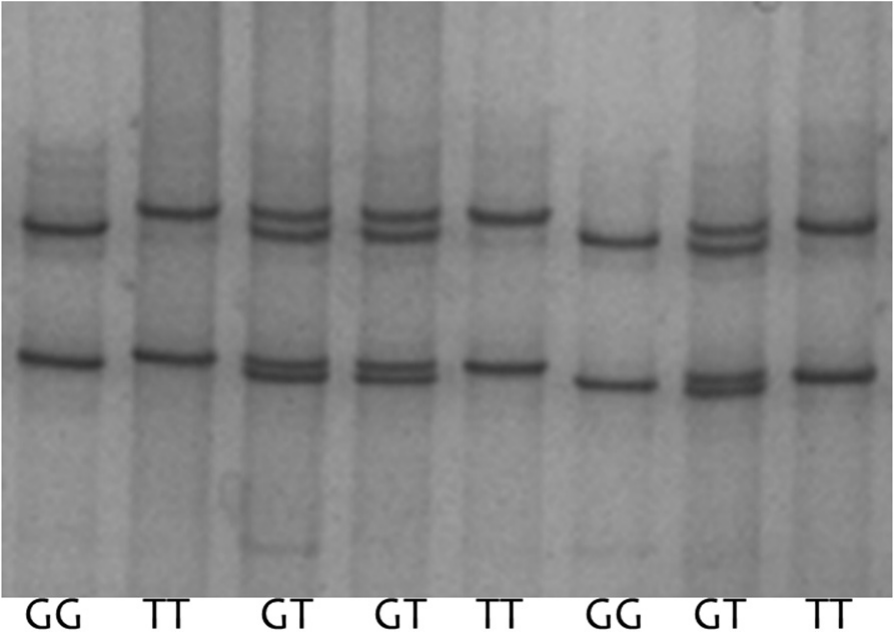
**Representative genotyping of *LHCGR *by SSCP followed by polyacrylamide gel electrophoresis.** A single mutation reveals three genotypes: four bands numbered 1, 2, 3 and 4 from bottom up in one lane make the GT genotype, No.1 and No.3 bands make the GG genotype, and No.2 and No.4 bands make the TT genotype.

**Figure 2:**
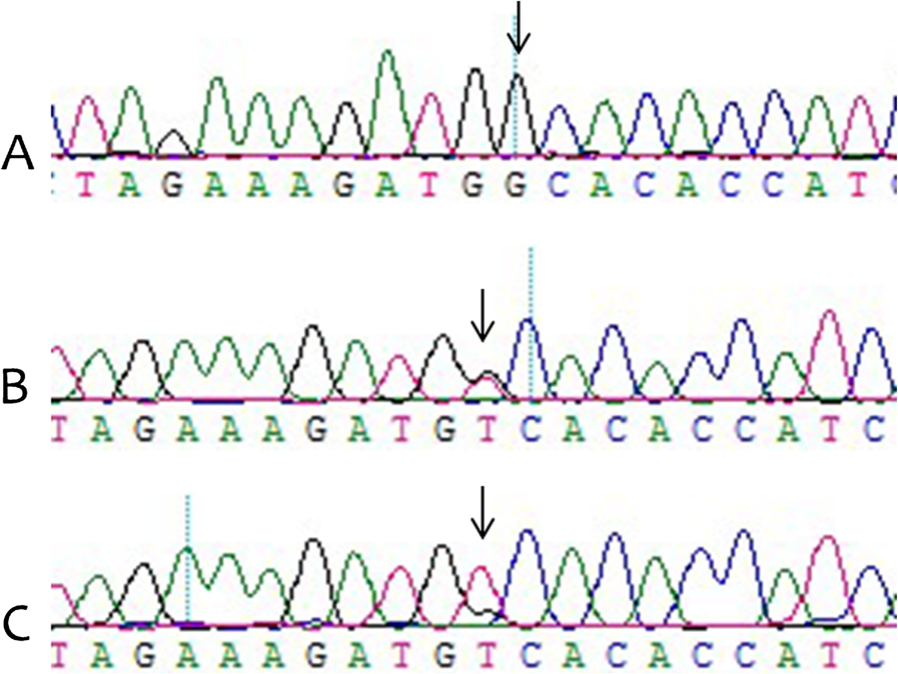
**A, B and C represent sequencing results of products with GG, GT and TT genotypes respectively.** Arrows indicate the mutation site.

After DNA sequencing, we found a single mutation (ss52050737) in the 1049 bp fragment amplified with primer set 1. Genotyping of the mutation was performed by SSCP with primer set 2, and the corresponding three genotypes were named GG, GT, and TT (Figure 1). Products with three genotypes were sequenced respectively for sake of avoiding the false positive of SSCP (Figure 2). The frequencies of the G and T alleles were 0.398 and 0.602, respectively, and the frequencies of the GG, GT, and TT genotypes were 0.150, 0.496, and 0.354, respectively.

### Association of genotypes with superovulation traits

The numbers of non-responders with the GG, GT, and TT genotypes were 2, 7, and 4 respectively. The results of association analysis between genotypes and superovulation traits are shown in Table 1. Heifers with the GG and GT genotypes had a significantly higher total number of ova (TNO) than those with the TT genotype (*P* < 0.05), and heifers with the GG genotype had a significantly higher number of transferable embryos (NTE) than those with GT and TT (*P* < 0.05). Heifers with the GG genotype had a smaller number of unfertilized ova (NUO) than those with the GT and TT genotypes, although only the difference between GG and GT was significant. There were no significant differences in the number of degenerate embryos (NDE) among the three genotypes.

**Table 1:** **Association of *LHCGR *genotypes with superovulation traits**

**Traits**	**GG (17)**^**a**^	**GT (56)**	**TT (41)**	**P value**
TNO	10.989 ± 1.517^b^	10.287 ± 0.889^b^	7.036 ± 1.057^c^	0.020
NTE	6.615 ± 1.020^b^	4.262 ± 0.686^bd^	3.246 ± 0.836^d^	0.008
NUO	1.518 ± 0.826^b^	3.445 ± 0.555^c^	2.479 ± 0.677^bc^	0.046
NDE	2.175 ± 0.866	2.086 ± 0.582	1.434 ± 0.710	0.557

^a^The number of samples is shown in parentheses. All data in the table are least square means ± SE. Different superscript letters (b-d) in the same row indicate significant differences (P < 0.05). TNO: total number of ova; NTE: number of transferable embryos; NUO: number of unfertilized ova; NDE: number of degenerate embryos.

## Discussion

The use of peripubertal donors in embryo transfer (ET) programs has been studied, and no differences in TNO or NTE have been found among heifers that were older than 10 months of age [24]. Furthermore, reproductive and lactational traits of heifers subjected to embryo transfer do not differ from their untreated full siblings [24]. Thus, the heifers in this study can be used as donors in embryo transfer programs without compromising their subsequent reproductive or lactational performance [24]. Hayakawa et al. (2009) compared the outcomes of superovulation and embryo transfer with sexed and unsexed sperm and observed no difference between the two groups for superovulation traits or pregnancy rates [25]. These observations are consistent with results of our previous study [26]. For AI in our study, sexed frozen-thawed sperm from different bulls was used randomly to inseminate heifers.

Heifers with the GG genotype had significantly higher TNO and NTE than those with the TT genotype, indicating that the G allele had a favorable, positive effect on these two superovulation traits. Three SNPs (ss52050737, ss52050738, and ss52050739) in exon 11 of *LHCGR* and their significant associations with variations in cattle fertility were described [22], and our results further demonstrated the SNP (ss52050737), in particular, was associated with a positive response to superovulation.

As a response to superovulatory treatment in cattle, more ovarian follicles initiate growth, granulosa cells proliferate and acquire follicle stimulating hormone receptors, and LHRs are induced, permitting the granulosa cells to later respond to LH [1]. Subsequently, several events occur: resumption of oocyte meiosis, transformation of the steroid enzyme complex from production of estrogen to production of progesterone, follicular rupture, and finally corpus luteum formation when serum LH levels increase during the preovulatory LH surge [1]. In cattle, moreover, LH may promote cytoplasmic maturation or competence of the oocyte as well as embryonic development after fertilization, both in vivo and in vitro [4,5,27,28]. The presence of LHRs in bovine oocytes, embryos, and blastocysts has been reported[5]. Bovine oviducts also express LHRs, and their activation results in increased synthesis of oviductal glycoprotein [29], which binds to embryos to enhance their development [30-34]. Thus, LH may enhance embryonic development through both direct and indirect mechanisms after binding to its receptor. As a G protein–coupled receptor, binding of the LHCGR allows dissociation of membrane-bound cognate G proteins that regulate phospholipase C, adenylyl cyclase, and ion channels, which in turn control cellular inositol phosphates, cAMP, Ca^2+^, and other secondary messengers [8]. Exon 11, in which SNP ss52050737 is located, encodes the intracellular domain of the LHCGR protein. Therefore, a missense mutation may change the structure of the intracellular region of the LHCGR and reduce the effect of LH to explain the functional relevance of this mutation to variation in response to superovulation and phenotype. However, the biological mechanism by which the mutation influences the function of LHCGR requires further investigation.

## Conclusions

The results of this study indicate that *LHCGR* is a potential marker for superovulation traits and can be used as a predictor for superovulation in Chinese Holstein heifers.

## Competing interests

The authors declare that they have no competing interests.

## Authors' contributions

Yong Yu participated in experimental design, data collection, analysis of the data and manuscript preparation; Yunwei Pang was responsible for research design, analysis of the data and draft the manuscript; Haichao Zhao helped for data collection and coordinated the research; Xiaoling Xu helped for analysis of the data; Zhonghong Wu helped for manuscript revising; Lei an help with data collection; Jianhui Tian contributed in experimental design and made the reasonable corrections on the manuscript. All authors read and approved the final manuscript.
